# Epidemiologie seelischen Wohlbefindens von Kindern und Jugendlichen in Deutschland. Ergebnisse aus 3 Studien vor und während der COVID-19-Pandemie

**DOI:** 10.1007/s00103-023-03720-5

**Published:** 2023-05-30

**Authors:** Franziska Reiß, Anne Kaman, Ann-Kathrin Napp, Janine Devine, Lydia Y. Li, Lisa Strelow, Michael Erhart, Heike Hölling, Robert Schlack, Ulrike Ravens-Sieberer

**Affiliations:** 1 Zentrum für Psychosoziale Medizin, Klinik für Kinder- und Jugendpsychiatrie, -psychotherapie und -psychosomatik, Universitätsklinikum Hamburg-Eppendorf, Martinistraße 52, 20246 Hamburg, Deutschland; 2 Alice Salomon Hochschule, Berlin, Deutschland; 3 Apollon Hochschule der Gesundheitswirtschaft, Bremen, Deutschland; 4 Robert Koch-Institut, Berlin, Deutschland

**Keywords:** Gesundheitsbezogene Lebensqualität, Psychische Gesundheit, BELLA-Studie, COPSY-Studie, HBSC-Studie, Health-related quality of life, Mental health, BELLA Study, COPSY Study, HBSC Study

## Abstract

**Hintergrund:**

Ein kontinuierliches bundesweites Gesundheitsmonitoring ist wichtig, um das Wohlbefinden von Kindern und Jugendlichen im Blick zu behalten und Entwicklungsverläufe abzubilden. Anhand der Ergebnisse von 3 ausgewählten epidemiologischen Studien werden Entwicklungen zum kindlichen Wohlbefinden der letzten 20 Jahre vorgestellt.

**Methodik:**

Datengrundlage bilden (1.) die bevölkerungsbezogene BEfragung zum seeLischen WohLbefinden und VerhAlten (BELLA-Studie, 2003–2017, *N* = 1500–3000), die ein Modul der KiGGS-Studie ist, (2.) die COrona und PSYche Studie (COPSY, 2020–2022, *N* = 1600–1700), die auf der BELLA-Studie aufbaut, und (3.) die internationale Health-Behaviour in School-aged Children Studie (HBSC, 2002–2018, *N* = 4300–7300). Das Wohlbefinden wurde bei 7‑ bis 17-Jährigen mittels der Indikatoren gesundheitsbezogene Lebensqualität (KIDSCREEN-10), Lebenszufriedenheit (Cantril Ladder) und psychische Auffälligkeiten (Strenghts and Difficulties Questionnaire (SDQ), Screen for Child Anxiety Related Emotional Disorders (SCARED) und Center for Epidemiological Studies Depression Scale for Children (CES-DC)) erfasst.

**Ergebnisse:**

Insgesamt zeigen Kinder und Jugendliche präpandemisch (2002–2018) eine konstant hohe gesundheitsbezogene Lebensqualität und eine hohe allgemeine Lebenszufriedenheit, die sich mit Beginn der COVID-19-Pandemie 2020 zunächst verschlechterte. 2 Jahre später zeigen sich Verbesserungen, die jedoch noch nicht das Ausgangsniveau erreichen. Psychische Auffälligkeiten, ängstliche und depressive Symptome nahmen mit Pandemiebeginn um bis zu 12 Prozentpunkte zu und zeigen auch 2 Jahre nach Pandemiebeginn noch höhere Werte als präpandemische Studien.

**Diskussion:**

Die Epidemiologie kindlichen Wohlbefindens bietet eine notwendige Datengrundlage, um den Unterstützungsbedarf von Kindern und Jugendlichen zu erfassen und auf dieser Basis Maßnahmen der Gesundheitsförderung, Prävention und Intervention zu entwickeln.

**Zusatzmaterial online:**

Zusätzliche Informationen sind in der Online-Version dieses Artikels (10.1007/s00103-023-03720-5) enthalten.

## Abstract (en)

**Background:**

Continuous nationwide health monitoring is important to track the well-being of children and adolescents and to map developmental trajectories. Based on the results of three selected epidemiological studies, developments in child well-being over the past 20 years are presented.

**Methods:**

Data are based on (1) the mental health module of the German National Health Interview and Examination Survey among Children and Adolescents (BELLA study, 2003–2017, *N* = 1500 to 3000), which is a module of the KiGGS study; (2) the COvid-19 and PSYchological Health Study (COPSY, 2020–2022, *N* = 1600–1700), which is based on the BELLA Study; and (3) the International Health-Behaviour in School-aged Children Study (HBSC, 2002–2018, *N* = 4300–7300). Well-being was assessed in 7‑ to 17-year-olds using indicators of health-related quality of life (KIDSCREEN-10), life satisfaction (Cantril Ladder), and mental health problems (Strenghts and Difficulties Questionnaire (SDQ), Screen for Child Anxiety Related Emotional Disorders (SCARED), and Center for Epidemiological Studies Depression Scale for Children (CES-DC)).

**Results:**

Overall, children and adolescents show consistently high health-related quality of life and high overall life satisfaction pre-pandemic (2002–2018), which initially worsened with the onset of the 2020 COVID-19-pandemic. Two years later, improvements are evident but have not yet reached baseline levels. Psychological problems, as well as symptoms of anxiety and depression, increased by up to 12 percentage points at the beginning of the pandemic and are still higher two years after the onset of the pandemic compared to pre-pandemic studies.

**Conclusion:**

The epidemiology of child well-being provides a necessary data basis to assess the support needs of children and adolescents and to use this as a basis for developing measures of health promotion, prevention, and intervention.

## Einleitung

Mit dem Ausbruch der COVID-19-Pandemie in Deutschland im Frühjahr 2020 ist deutlich geworden, wie wichtig umfassende Daten zur gesundheitlichen Situation von Kindern und Jugendlichen sind und wie sehr gesellschaftspolitische und gesundheitsbezogene Maßnahmen mit diesen assoziiert sind. Dabei gilt es nicht nur, aktuelle Daten zu generieren, um beispielsweise die Auswirkungen der pandemischen Schutzmaßnahmen auf die Gesundheit und das Wohlbefinden von Kindern und Jugendlichen zu untersuchen, sondern auch Entwicklungsverläufe abzubilden sowie Risikogruppen und gesundheitliche Ressourcen zu identifizieren.

In der Epidemiologie, der Wissenschaft von der Verbreitung, den Ursachen und Folgen von gesundheitsbezogenen Zuständen und Ereignissen in Bevölkerungen, ist der Begriff Wohlbefinden bislang nicht einheitlich definiert. Die Weltgesundheitsorganisation (WHO) definiert Gesundheit im Allgemeinen als einen Zustand des vollständigen körperlichen, seelischen und sozialen Wohlbefindens [1]. In diesem Beitrag werden zentrale Indikatoren seelischen Wohlbefindens berücksichtigt, die ein gesundes Aufwachsen von Kindern und Jugendlichen beeinflussen und sowohl positiv konnotiert sind, wie die „gesundheitsbezogene Lebensqualität“ und die „subjektive Lebenszufriedenheit“ [2], als auch negativ konnotiert sind, wie „psychische Auffälligkeiten“ [3]. Diese zählen auch zu den zentralen Gesundheitsindikatoren, wie sie von der WHO für die Etablierung eines Informations- und Monitoringsystems benannt werden [4].

Bisher hat die Epidemiologie des seelischen Wohlbefindens eine Vielzahl empirischer Studien hervorgebracht. So wird die gesundheitsbezogene Lebensqualität von Kindern und Jugendlichen in einer europäischen Vergleichsstudie aus dem Jahr 2005 als hoch eingeschätzt, wobei etwa 70 % eine sehr gute Lebensqualität berichten [5]. Eine hohe allgemeine Lebenszufriedenheit wird auch im nationalen und internationalen Vergleich berichtet, wie Trendanalysen aus den Jahren 2002 bis 2010 mit mehr als 30 Ländern zeigen [6]. Zudem werden die Prävalenzen psychischer Erkrankungen, alters- und geschlechtsspezifische Verteilungen und Persistenzraten vom Kindes- und Jugendalter bis ins Erwachsenenalter epidemiologisch erfasst [7, 8]. Die mittlere Prävalenzrate psychischer Störungen wird in einer Metaanalyse internationaler Studien im Beobachtungszeitraum von 1985–2012 auf 13,4 % (KI 95 %: 11,3–15,9; [9]) und im deutschsprachigen Raum von 1953–2007 auf 17,6 % (KI 95 %: 15,66–19,51) geschätzt [10]. In den genannten Beobachtungszeiträumen finden diese Metaanalysen keine Zunahme psychischer Störungen bei Kindern und Jugendlichen, wobei einschränkend angemerkt sei, dass nicht das gesamte Spektrum psychischer Störungen im Kindes- und Jugendalter in Überblicksarbeiten erfasst wird und die Studien bereits etwas älter sind.

Aktuelle internationale Überblicksarbeiten zeigen, dass mit dem Beginn der COVID-19-Pandemie psychische Auffälligkeiten bei Heranwachsenden zugenommen haben [11]. Psychische Erkrankungen im Kindes- und Jugendalter sind bedeutsam, da sie häufig bis ins Erwachsenenalter persistieren und somit nicht nur mit einem hohen, langen Leidensdruck der Betroffenen und Beeinträchtigungen der Bildungs- und Berufschancen sowie der Partizipation verbunden sind, sondern auch mit hohen gesundheitsökonomischen Kosten. Zudem ist die Epidemiologie seelischen Wohlbefindens aktuell von hoher Public-Health-Relevanz, da gesellschaftliche Krisen, wie z. B. die COVID-19-Pandemie, die Klimakrise und der Krieg in der Ukraine, auch Kinder und Jugendliche beschäftigen und Auswirkungen auf deren gesundheitliches Wohlbefinden haben. Regelmäßige Datenerhebungen wie die Trendstudie „Jugend in Deutschland“ [12, 13] zeigen, wie sehr diese Krisen Heranwachsende belasten und Zukunftsängste sowie Unsicherheit hervorrufen können.

Eine Besonderheit der Erhebung kindlichen Wohlbefindens ist, dass bei jüngeren Kindern Informationen über die Eltern oder andere Bezugspersonen erfragt werden müssen. Bei altersentsprechenden Fragebögen können jedoch Kinder auch schon ab dem Alter von 8 Jahren eine verlässliche Selbstauskunft zu ihrer Gesundheit geben [14]. In Abgrenzung zu anderen Datenquellen, wie beispielsweise Routineversorgungsdaten der Krankenkassen, bieten epidemiologische Studien den Vorteil, den selbstberichteten Gesundheitszustand zu erfassen. Zur Erfassung der verschiedenen Dimensionen von Wohlbefinden in der Altersspanne von 3 bis 17 Jahren gibt es gut validierte und international etablierte Messinstrumente.

Epidemiologische Studien, welche eine fortlaufende und systematische Erhebung, Analyse, Interpretation und Berichterstattung von gesundheitsbezogenen Daten ermöglichen, sind ein wichtiger Bestandteil der Public-Health-Forschung und Surveillance [15] und zählen zu den Kernpunkten einer Public-Health-Strategie für Deutschland [16], wie sie seit 2018 am Robert Koch-Institut aufgebaut wird [17]. Ein kontinuierliches Monitoring des seelischen Wohlbefindens der Heranwachsenden kann somit Entwicklungen aufzeigen und besonders belastete Kinder und Jugendliche sowie hilfreiche Ressourcen identifizieren. Auf diese Weise können Zielgruppen für Maßnahmen identifiziert, evidenzbasierte Präventions- und Interventionsangebote entwickelt und in ihrer Implementierung begleitet werden.

Ziel unseres Beitrags ist, einen Überblick ausgewählter Ergebnisse aus 3 epidemiologischen Studien zum kindlichen Wohlbefinden im Verlauf der vergangenen 2 Jahrzehnte zu geben und somit den Zeitraum vor und während der COVID-19-Pandemie abzubilden. Dazu zählt die BELLA-Studie (BEfragung zum seeLischen WohLbefinden und VerhAlten), welche das Zusatzmodul zur psychischen Gesundheit der vom Robert Koch-Institut durchgeführten Studie zur Gesundheit von Kindern und Jugendlichen in Deutschland (KiGGS) ist. Darauf aufbauend wurde mit Beginn der COVID-19-Pandemie die COPSY-Studie (COrona und PSYche) entwickelt, die seit dem Frühjahr 2020 kontinuierlich Eltern zur psychischen Gesundheit ihrer Kinder sowie auch die Kinder selbst befragt. Internationale vergleichbare Daten der HBSC-Studie (Health Behaviour in School-aged Children) werden ebenfalls berücksichtigt.

Folgende Fragestellungen wurden untersucht: Wie haben sich 1) die gesundheitsbezogene Lebensqualität, 2) die selbstberichtete Lebenszufriedenheit sowie 3) psychische Auffälligkeiten im Allgemeinen sowie Ängstlichkeit und depressive Symptome im Besonderen bei Kindern und Jugendlichen in den vergangenen 20 Jahren in Deutschland entwickelt?

## Methoden

### Studiendesign und Stichprobe

Die Datengrundlage bilden die bundesweiten bevölkerungsbezogenen Längsschnittstudien BELLA und COPSY [18, 19]. Beide Studien sind als kombinierter Längs- und Querschnittansatz konzipiert, d. h. es werden sowohl Teilnehmende wiederholt befragt (Längsschnitt) als auch zu jedem Befragungszeitpunkt neue Teilnehmende hinzugewonnen (repräsentativer Querschnitt), um Ausfälle durch ausscheidende Teilnehmende zu kompensieren und die Repräsentativität zu erhalten. Die Stichproben der BELLA-Studie und der COPSY-Studie wurden gewichtet, um in den wesentlichen Merkmalen der Bevölkerungsstruktur von Familien mit Kindern in Deutschland gemäß dem aktuellen Mikrozensus zu entsprechen.

Für die präpandemische BELLA-Studie wurden im Zeitraum von 2003 bis 2017 zu insgesamt 5 Messzeitpunkten Familien befragt, deren Kinder zwischen 7 und 17 Jahre alt waren. Die Befragung erfolgte von 2003 bis 2012 per Papierfragebogen und computerassistierter Telefoninterviews (CATI) sowie von 2014 bis 2017 per Onlinebefragung. Für die COPSY-Studie wurden im Zeitraum von Mai 2020 bis Oktober 2022 zu 5 Messzeitpunkten Familien per Onlineerhebung befragt, deren Kinder zwischen 7 und 17 Jahre alt waren. In der BELLA- und COPSY-Studie wurden Angaben für 7‑ bis 17-Jährige von jeweils einem Elternteil sowie ab 11 Jahren zusätzlich Angaben im Selbstbericht erhoben. Weitere Informationen zu Design, Methodik und Ergebnissen der BELLA- und COPSY-Studie finden sich bei Otto et al. [18] und Ravens-Sieberer et al. [19].

Die dritte Datengrundlage bildet die Schulbefragung im Rahmen der HBSC-Studie, welche von 2002 bis 2018 im vierjährigen Turnus bundesweit 11-, 13- und 15-jährige Schülerinnen und Schüler zu ihrer körperlichen und psychischen Gesundheit sowie dem Gesundheitsverhalten befragt. Die HBSC-Studie wird von der WHO unterstützt und findet mittlerweile in 51 Ländern gleichzeitig statt. Die deutschen Stichproben basieren auf einer Zufallsauswahl von Schulklassen aller öffentlichen Schulen. Die letzte Erhebung fand im Schuljahr 2021/2022, wahlweise mittels eines schriftlichen oder eines Onlinefragebogens, statt. Weitere Informationen zu Design, Methodik und Ergebnissen der HBSC-Studie finden sich bei Ottova et al. [20].

In allen Studien wurden soziodemographische Angaben zu Geschlecht, Alter und Migrationshintergrund der Kinder im Selbst- und/oder Elternbericht erfasst.

### Indikatoren kindlichen Wohlbefindens

Das mehrdimensionale Konstrukt der *gesundheitsbezogenen Lebensqualität* wurde in der BELLA- und in der COPSY-Studie mithilfe des „KIDSCREEN-10“-Instrumentes erfasst. Dies ist ein international validiertes Maß, welches simultan in mehr als 15 Ländern im Rahmen der EU-geförderten europäischen KIDSCREEN-Studie entwickelt wurde [21]. Der KIDSCREEN-10 wurde für die Altersgruppe der 7‑ bis 10-Jährigen als Elternbericht sowie bei den 11- bis 17-Jährigen als Selbstbericht erfasst und setzt sich aus 10 Items zusammen. Die 5‑stufige Likert-Skala umfasst die Antwortmöglichkeiten von *nie* bis *immer* bzw. von *überhaupt nicht* bis *sehr*. Die Antworten werden aufsummiert und in T‑Werte transformiert. Für die Normstichprobe ist der Mittelwert auf 50 festgelegt und die Standardabweichung auf ± 10. Ein höherer T‑Wert bildet eine höhere gesundheitsbezogene Lebensqualität ab und ein niedrigerer T‑Wert eine niedrigere.

Die Bewertung der *Lebenszufriedenheit* wurde in der HBSC- und in der COPSY-Studie von Kindern und Jugendlichen ab 11 Jahren im Selbstbericht mittels der Cantril Ladder erfasst [22]. Auf einer 11-stufigen visuellen Analogskala in Form einer Leiter geben die Befragten an, auf welcher Stufe sie ihr derzeitiges Leben verorten. Das obere Ende der Leiter bezeichnet dabei *das beste denkbare Leben* (10 Punkte) und das untere Ende *das schlechteste denkbare Leben* (0 Punkte). Ein Wert von mindestens 6 wird als höhere Lebenszufriedenheit definiert.

*Psychische Auffälligkeiten* wurden in der BELLA- und COPSY-Studie mittels des Gesamtproblemwerts des etablierten „Strenghts and Difficulties Questionnaire“ (SDQ) erfasst. Dieser beinhaltet die 4 Subskalen Verhaltensprobleme, emotionale Probleme, Hyperaktivität und Probleme mit Gleichaltrigen [23] und wurde für die 7‑ bis 17-Jährigen im Elternbericht (BELLA-Studie 2014–2017 und COPSY-Studie 2020–2022) sowie zusätzlich für 11- bis 17-Jährige im Selbstbericht (BELLA-Studie 2003–2012) erhoben. In der BELLA-Erhebung 2009–2012 wurde der Elternbericht für Kinder ab 3 Jahren erhoben. Eine Kategorisierung der Kinder- und Jugendlichen in *unauffällig* und *auffällig bzw. grenzwertig auffällig* erfolgte anhand der deutschen Cut-off-Werte [24].

*Symptome von Ängstlichkeit* wurden in der BELLA- und in der COPSY-Studie mittels der deutschen Version des „Screen for Child Anxiety Related Emotional Disorders“ (SCARED) für 11- bis 17-Jährige im Selbstbericht erfasst [25, 26]. Die Kategorisierung des Gesamtsummenwerts des SCARED in *unauffällig* und *auffällig* wurde gemäß den publizierten Cut-off-Werten vorgenommen [25].

*Depressive Symptome* wurden in der BELLA- und in der COPSY-Studie mittels der „Center for Epidemiological Studies Depression Scale for Children“ (CES-DC; [27]) in der deutschen Übersetzung [28] für 11- bis 17-Jährige im Selbstbericht erfasst. Erhoben wurden die 4 Subskalen körperliche Probleme, depressive Gefühle und positive Gefühle sowie interpersonelle Probleme. Zur Kategorisierung in *unauffällig* versus *auffällig,* basierend auf dem Gesamtwert der CES-DC, wurde der entsprechend publizierte Cut-off verwendet [29].

### Statistische Analysen

Die Datenauswertung erfolgte mittels deskriptiver Analysen. Für die Lebensqualität (KIDSCREEN-10) wurden gemittelte T‑Werte berechnet, für psychische Auffälligkeiten (SDQ) sowie Symptome von Depression (CES-DC) und Ängstlichkeit (SCARED) wurden relative Häufigkeiten berechnet. Mittelwerte wurden ebenfalls für die Lebenszufriedenheit (Cantril Ladder Scale) von Kindern und Jugendlichen erhoben. Alle Analysen erfolgten in IBM SPSS Version 27.

## Ergebnisse

### Stichprobenbeschreibung

An der BELLA-Studie nahmen zu den einbezogenen Messzeitpunkten Kinder und Jugendliche im Alter von 7 bis 17 Jahren sowie deren Eltern teil (BELLA 2003–2006: *N* = 2863; BELLA 2014–2017: *N* = 1580). Zudem wurden auch Daten der jüngeren Kinder ab 3 Jahren betrachtet (BELLA 2009–2012: *N* = 2411).

In der COPSY-Studie wurden zu den 5 Erhebungszeitpunkten zwischen Frühsommer 2020 und Herbst 2022 jeweils etwa 1600 bis 1700 Eltern von 7‑ bis 17-Jährigen sowie über 1000 Kinder und Jugendliche ab 11 Jahren befragt (COPSY I 05/06 2020: *N* = 1586 Eltern/1040 Kinder; COPSY II 12/2020–01/2021: *N* = 1625/1077; COPSY III 09/10 2021: *N* = 1618/1139; COPSY IV 02/2022: *N* = 1668/1079; COPSY V 09/10 2022: *N* = 1701/1085).

An der HBSC-Befragung nahmen je Messzeitpunkt Kinder und Jugendliche im Alter von 11, 13 und 15 Jahren teil (HBSC 2002: *N* = 5650; HBSC 2006: *N* = 7274; HBSC 2010: *N* = 5005; 2014: *N* = 5961; HBSC 2018: *N* = 4347; [20, 30, 31]). Ein Überblick über die soziodemographischen Merkmale der Stichproben befindet sich im zusätzlichen Onlinematerial (Tabelle Z1).

### Gesundheitsbezogene Lebensqualität

**Figure 1:**
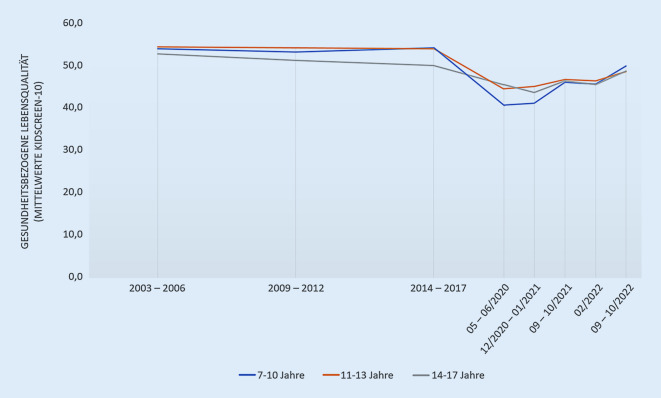

Insgesamt wurde die gesundheitsbezogene Lebensqualität von Kindern und Jugendlichen in Deutschland zwischen 2003 und 2017 so eingeschätzt, dass der Mittelwert im Normbereich (um M = 50) lag (Abb. 1). Das bedeutet, dass sich die Kinder und Jugendlichen präpandemisch über 2 Jahrzehnte lang im Durchschnitt fit und wohl gefühlt haben, in Bezug auf ihr Familienleben und die Beziehung zu Gleichaltrigen zufrieden waren und auch in der Schule gut zurechtgekommen sind [32]. Zu Beginn der Pandemie (Mai/Juni 2020) zeigte sich bei Kindern und Jugendlichen ein Abfall der gesundheitsbezogenen Lebensqualität, der sich bis September/Oktober 2022 noch nicht auf präpandemische Werte erholte (siehe Abb. 1). Insgesamt hatten über den gesamten Verlauf die 14- bis 17-jährigen Jugendlichen (Selbstbericht) eine etwas schlechtere Lebensqualität, als die 7‑ bis 10-jährigen Kinder (Elternbericht). Demnach lag der mittlere T‑Wert (in der BELLA-Studie) bei den 7‑ bis 10-jährigen Kindern zwischen 2003 und 2017 bei etwa 54, im Mai/Juni 2020 lag er (in der COPSY-Studie) bei etwa 40. Im weiteren Pandemieverlauf nahm die gesundheitsbezogene Lebensqualität wieder zu, erreichte aber mit einem mittleren T‑Wert von etwa 50 im September/Oktober 2022 noch nicht wieder das präpandemische Niveau. Die HBSC-Studie zeigte zudem, dass Mädchen eine geringere gesundheitsbezogene Lebensqualität hatten als Jungen [33].

### Lebenszufriedenheit

**Figure 2:**
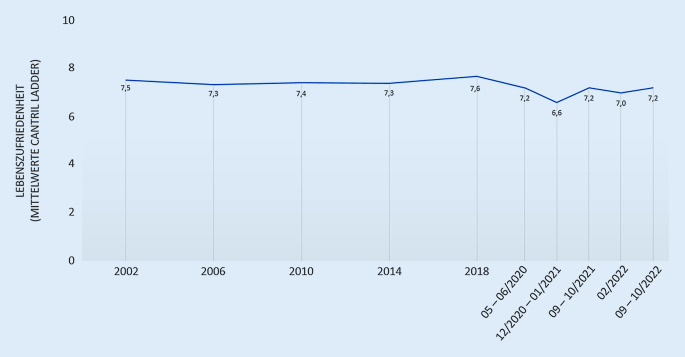

Die Ergebnisse für die Lebenszufriedenheit von Kindern und Jugendlichen sind in Abb. 2 dargestellt. Auf einer Skala von 0 Punkten (das „*schlechteste denkbare Leben*“) bis 10 Punkten (das „*beste denkbare Leben*“) schätzten Kinder und Jugendliche in Deutschland ihre Lebenszufriedenheit präpandemisch im Mittel mit 7,5 Punkten (HBSC-Befragung 2002) als relativ hoch ein und verblieben über einen Zeitraum von anderthalb Jahrzehnten in etwa auf diesem Niveau (HBSC-Befragung 2018). Das bedeutet, dass sie mit ihrem Leben im Allgemeinen recht zufrieden waren. Ergebnisse der COPSY-Studie zeigten, dass mit dem Beginn der COVID-19-Pandemie die Einschätzung der Lebenszufriedenheit mit einem Mittelwert von 7,2 Punkten erstmalig nach 1,5 Jahrzehnten geringer ausfiel und im Lockdown weiter auf 6,6 Punkte (Dezember 2020 bis Januar 2021) sank. 2 Jahre nach Pandemiebeginn zeigen Kinder und Jugendliche in Deutschland zwar eine deutlich bessere Lebenszufriedenheit im Vergleich zur Hochphase der Pandemie, die präpandemischen Werte werden jedoch nicht erreicht.

### Psychische Auffälligkeiten

**Figure 3:**
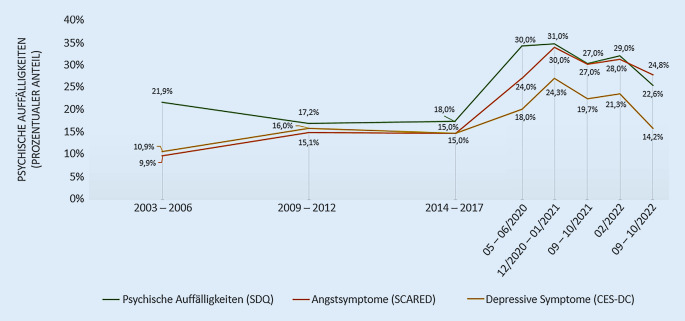

Die Ergebnisse zu psychischen Auffälligkeiten im Allgemeinen sowie Symptomen von Ängstlichkeit und Depressivität im Besonderen sind in Abb. 3 dargestellt. Während im Erhebungszeitraum 2003 bis 2017 über 1,5 Jahrzehnte die Prävalenzen psychischer Auffälligkeiten von 22 % (2003 bis 2006) auf 18 % (2014 bis 2017) rückläufig waren, kam es zu Beginn bzw. im ersten Jahr der COVID-19-Pandemie zu einem deutlichen Anstieg psychischer Auffälligkeiten auf etwa 30 %. Im Laufe des Jahres 2021 bis zum Frühjahr 2022 lag die Prävalenz psychischer Auffälligkeiten stabil bei etwa 27–29 %. Seither ist der Anteil der Kinder und Jugendlichen, die Hinweise auf psychische Auffälligkeiten zeigen, rückläufig auf bis zu 23 % (September/Oktober 2022), hat jedoch das präpandemische Niveau noch nicht wieder erreicht.

Symptome von Ängstlichkeit bei Kindern und Jugendlichen waren im Zeitraum zwischen der ersten BELLA-Befragung (2003 bis 2006) von 10 % auf 15 % gestiegen (2009 bis 2012) und hatten sich dann auf diesem Niveau bis zur letzten BELLA-Befragung (2014 bis 2017) mit einer Prävalenz von 15 % stabilisiert. Mit dem Beginn der COVID-19-Pandemie stiegen Symptome von Ängstlichkeit auf bis zu 30 % (Dezember 2020 bis Januar 2021) und waren damit im Vergleich zu der Zeit vor der Pandemie doppelt so hoch. Im Jahr 2021 bis zum Frühjahr 2022 zeigte sich eine weitgehende Stabilisierung der Ängstlichkeitswerte bei 27–28 % und ist seither rückläufig auf etwa 25 %, womit das präpandemische Niveau jedoch noch nicht wieder erreicht wird.

Depressive Symptome bei Kindern und Jugendlichen zeigten im präpandemischen Zeitraum einen ähnlichen Verlauf wie Symptome von Ängstlichkeit. So konnte im ersten Befragungszeitraum der BELLA-Studie von 2003 bis 2006 bis zum Befragungszeitpunkt 2009 bis 2012 ein Anstieg depressiver Symptome von 11 % auf 16 % beobachtet werden. Anschließend blieben die Prävalenzen bis zur nächsten BELLA-Befragung (2014 bis 2017) mit 15 % auf einem ähnlichen Niveau stabil. Mit dem Beginn der COVID-19-Pandemie zeigte sich dann ein Anstieg depressiver Symptome auf bis zu 24 % (Dezember 2020 bis Januar 2021). Im Jahr 2021 bis Frühjahr 2022 zeigte sich eine relative Konstanz der Depressionssymptome bei 20 % und in der letzten COPSY-Befragung (September/Oktober 2022) dann schließlich ein Rückgang auf präpandemisches Niveau (14 %).

## Diskussion

Der Beitrag gibt einen Überblick über den Verlauf des seelischen Wohlbefindens von Kindern und Jugendlichen in Deutschland anhand von Ergebnissen dreier bundesweiter epidemiologischer Studien der vergangenen 20 Jahre.

Nach 1–2 präpandemischen Dekaden von konstant guter gesundheitsbezogener Lebensqualität, Lebenszufriedenheit und einem leichten Rückgang psychischer Auffälligkeiten von Kindern und Jugendlichen zeigten sich zu Beginn der COVID-19-Pandemie erhebliche Verschlechterungen. Auch wenn sich das seelische Wohlbefinden der Kinder und Jugendlichen nach fast 3 Jahren Pandemiegeschehen wieder deutlich verbessert hat, konnte das präpandemische Niveau im Herbst 2022 – außer bei depressiven Symptomen – noch nicht wieder erreicht werden.

Die verminderte gesundheitsbezogene Lebensqualität von Kindern und Jugendlichen infolge der COVID-19-Pandemie findet sich auch in anderen Studien [34–36]. Eine signifikante Verschlechterung der körperlichen und psychischen Lebensqualität von Kindern und Jugendlichen im Lockdown wird insbesondere bei Heranwachsenden mit einem niedrigen sozioökonomischen Status berichtet [19, 35, 37–39]. Der im Rahmen dieser Studie gezeigte Abfall der gesundheitsbezogenen Lebensqualität zu Beginn der Pandemie vor allem bei älteren Kindern und Jugendlichen deckt sich mit Ergebnissen vorheriger Studien [40, 41]. Die HBSC-Studie zeigte zudem, dass Mädchen eine geringere gesundheitsbezogene Lebensqualität hatten als Jungen [33], was in Übereinstimmung mit anderen Studien steht [6, 42].

Nachdem die Lebenszufriedenheit von Kindern und Jugendlichen lange Zeit vor der Pandemie hoch war, nahm sie mit Beginn der COVID-19-Pandemie und im ersten Pandemiejahr deutlich ab, wie auch andere internationale Studien belegen [43–45]. Studien aus der Zeit vor der Pandemie zeigen, dass Kinder und Jugendliche aus Deutschland im internationalen Vergleich ähnliche Lebenszufriedenheitswerte wie die europäischen Nachbarländer aufwiesen [6], wobei Mädchen in allen Ländern eine signifikant geringere Lebenszufriedenheit zeigten als Jungen [6, 46, 47]. Ein etwas älteres Literatur-Review von Proctor et al. verweist anhand von 141 empirischen Studien auf die Wechselbeziehungen von Lebenszufriedenheit und psychischer und körperlicher Gesundheit sowie der Umwelt und dem Verhalten von Kindern und Jugendlichen. Die Untersuchung dieser Wechselbeziehungen wäre auch mit den vorliegenden Studiendaten möglich und interessant für zukünftige Forschungsarbeiten.

Nachdem die psychischen Auffälligkeiten über knapp 2 Jahrzehnte in Deutschland bei Kindern und Jugendlichen leicht rückläufig waren, zeigte sich zu Beginn der COVID-19-Pandemie ein deutlicher Anstieg auf bis zu 31 % im zweiten strengen bundesweiten Lockdown im Winter 2020/2021, der mehrmonatig mit weitreichenden Restriktionen verbunden war (u. a. Schulschließungen, Kontaktbeschränkungen, geschlossene Freizeiteinrichtungen). Dies steht in Einklang mit zahlreichen anderen deutschen Studien, wie ein Rapid-Review über 39 Publikationen zeigt [48]. Die Ergebnisse der COPSY-Studie zeigten darüber hinaus einen Anstieg von Ängstlichkeit und Depressivität im ersten Jahr der COVID-19-Pandemie und einen zögerlichen Abfall dieser Symptome im Verlauf der Pandemie. Die Ergebnisse ähneln internationalen Überblicksarbeiten, welche eine annähernde Verdoppelung von Depressionen und Angststörungen auf 21–29 % berichten [49, 50]. Dabei waren, ähnlich wie bei den Ergebnissen zur Lebensqualität, insbesondere Mädchen und ältere Kinder und Jugendliche häufiger betroffen.

In einer Studie von Zok und Roick (2022) zu pandemiebedingten Gesundheitsveränderungen berichteten Mütter von 3‑ bis 12-jährigen Kindern deutlich häufiger von Verschlechterungen der seelischen Gesundheit (35 % der Befragten) im Vergleich zu Verschlechterungen der körperlichen Gesundheit (16 % der Befragten; [37]). Aber auch positive Auswirkungen der COVID-19-Pandemie wurden berichtet. So hoben Eltern von Kindern im Alter von 3 bis 12 Jahren ein stärkeres Zusammengehörigkeitsgefühl in der Familie, neue Hobbys und eine höhere Selbstständigkeit des Kindes hervor.

Einschränkend ist darauf hinzuweisen, dass die 3 hier einbezogenen Studien keine Aussagen zu Kausalbeziehungen zulassen. Auch die zeitlichen Entwicklungen und Veränderungen bei der Lebenszufriedenheit, der gesundheitsbezogenen Lebensqualität und der psychischen Gesundheit von Kindern und Jugendlichen, unabhängig von der COVID-19-Pandemie, können nicht abschließend geklärt werden. Zudem wurde bei den Angaben zu psychischen Auffälligkeiten, Symptomen von Ängstlichkeit und Depression keine Differenzierung nach Altersgruppen bzw. nach Selbst- und Fremdauskunft vorgenommen.

Die Stärke des Beitrags besteht in der Zusammenfassung bevölkerungsbasierter gesamtdeutscher Ergebnisse anhand dreier epidemiologischer Studien (BELLA-, COPSY-, HBSC-Studie) mit insgesamt beachtlichen Stichprobengrößen und einer hohen wissenschaftlichen Qualität im Studiendesign und der angewandten Methodik über einen Zeitraum von 2 Jahrzehnten. Die stringente Anwendung der gleichen Fragebögen über die verschiedenen Studien ermöglicht die Vergleichbarkeit über diesen langen Zeitraum. Der Beitrag zeigt, wie umfassend sich eine gesundheitsbezogene gesellschaftliche Krise wie die COVID-19-Pandemie auf das seelische Wohlbefinden von Kindern auswirkt.

Aktuelle Studien geben deutliche Hinweise darauf, dass gesellschaftliche, umweltbezogene und politische Krisen zunehmend komplexe Auswirkungen auf die Gesundheit und Entwicklung haben, wobei Kinder und Jugendliche durch die direkten und indirekten Folgen mehrfach betroffen sind (z. B. körperlich, kognitiv, sozial-emotional; [51]). Deutsche sowie internationale Studien verweisen auf die negativen psychischen Auswirkungen neuer, aktueller Krisen [13, 52]. Zu den emotionalen Reaktionen zählen Ängste, aber auch Wut, Hilflosigkeit, Frustration und Trauer [52, 53]. Für das gesundheitliche Wohlbefinden haben diese insbesondere dann negative Konsequenzen, wenn sie über einen längeren Zeitraum anhalten. Sie sollten hinsichtlich von Prävention und Intervention stärker in den Blick genommen werden [51]. Eine umfassende, kontinuierliche Erhebung epidemiologischer Daten ist auch zukünftig unabdingbar. So empfehlt der Deutsche Ethikrat, dass die Forschung über die Folgen von Maßnahmen zur Bewältigung gesellschaftlicher Krisen allgemein gefördert werden sollte, um Angebote der Prävention, Beratung, Diagnostik, Therapie und Rehabilitation evidenzbasiert zu verbessern [54].

## Fazit und Empfehlungen

Epidemiologische Studien zum kindlichen Wohlbefinden schaffen die Datengrundlage für eine regelmäßige Dokumentation des Gesundheitszustandes in der Allgemeinbevölkerung, um (negative) Entwicklungen aufzuzeigen, Bedarfe zu erkennen und Interventionsmaßnahmen anzustoßen. Der Transfer der Forschungsergebnisse, von Wissenschaft und Forschung hin zu den Akteuren aus Politik und Praxis, ist hierbei von zentraler Bedeutung. Dabei gilt es, ein (öffentliches) Problembewusstsein zu schaffen und zu einer Verbesserung der gesundheitlichen Lage von Kindern und Jugendlichen beizutragen. Rückblickend werden beispielsweise die Maßnahmen zur Pandemiebekämpfung in Bezug auf das Wohlbefinden von Kindern und Jugendlichen kritisiert; z. B. bemängelt der Deutsche Ethikrat, dass die Belastungen und die psychische Gesundheit der jüngeren Generation in der Pandemie nicht ausreichend beachtet wurden. In Krisensituationen sollte die Generationengerechtigkeit beachtet werden, sodass transgenerationelle Solidarität nicht einseitig zu Lasten der jüngeren Generation gehe.

Angesichts vieler singulärer epidemiologischer Forschungsarbeiten wären eine Verstetigung langfristiger – auch schulbasierter, internationaler Studien (wie HBSC) – sowie eine breite nationale Planung und Vernetzung von Studien wünschenswert, um evidenzbasierte Daten auch zukünftig zu sammeln und Ergebnisse zeitnah generieren zu können. Epidemiologische Studien sollten dazu genutzt werden, um belastete Kinder sowie Ressourcen zu identifizieren. Somit können Zielgruppen für Maßnahmen benannt und bei der Setzung gesundheitspolitischer Ziele priorisiert berücksichtigt werden.

Wissenschaftsgremien wie der ExpertInnenrat der Bundesregierung empfehlen, sowohl die primäre als auch die sekundäre Krankheitslast unter spezifischer Berücksichtigung von Kindern und Jugendlichen wissenschaftlich zu erfassen und langfristig Fördermittel für die Führung eines kontinuierlichen bundesweiten epidemiologischen Gesundheits- und Maßnahmen-Monitorings bereitzustellen [55]. Erste bundesweite Forschungszusammenschlüsse wie das Netzwerk Universitätsmedizin (NUM) bieten beispielsweise den strukturellen Rahmen, um Kompetenzen und Ressourcen zu bündeln und Synergien gewinnbringend zu nutzen. Das Teilprojekt coverCHILD [56] setzt sich z. B. mit der Situation von Kindern und Jugendlichen während der COVID-19-Pandemie auseinander und erforscht, wie Heranwachsende in künftigen Krisen besser geschützt werden können.

## Supplementary Information





## References

[1] Weltgesundheitsorganisation (1948). Constitution of the World Health Organization.

[2] Helseth S, Haraldstad K, Michalos AC (2014). Child well-being. Encyclopedia of quality of life and well-being research.

[3] Pollard EL, Lee PD (2003). Child well-being: a systematic review of the literature. Soc Indic Res.

[4] World Health Organization (2021) Strengthening population health surveillance: a tool for selecting indicators to signal and monitor the wider effects of the COVID-19 pandemic. In: World Health Organization. Regional Office for Europe, Copenhagen. https://apps.who.int/iris/handle/10665/340720. Zugegriffen: 16. Jan. 2023

[5] Bisegger C, Cloetta B, von Bisegger U, Abel T, Ravens-Sieberer U, European Kidscreen Group (2005). Health-related quality of life: gender differences in childhood and adolescence. Soz Präventivmed.

[6] Cavallo F, Dalmasso P, Ottova-Jordan V (2015). Trends in life satisfaction in European and North-American adolescents from 2002 to 2010 in over 30 countries. Eur J Public Health.

[7] Fuchs M, Karwautz A (2017). Epidemiologie psychischer Störungen bei Kindern und Jugendlichen: Eine narrative Übersichtsarbeit unter Berücksichtigung österreichischer Daten. Neuropsychiatrie.

[8] Ihle W, Esser G (2002). Epidemiologie psychischer Störungen im Kindes- und Jugendalter. Psychol Rundsch.

[9] Polanczyk GV, Salum GA, Sugaya LS, Caye A, Rohde LA (2015). Annual research review: a meta-analysis of the worldwide prevalence of mental disorders in children and adolescents. J Child Psychol Psychiatry.

[10] Barkmann C, Schulte-Markwort M (2012). Prevalence of emotional and behavioural disorders in German children and adolescents: a meta-analysis. J Epidemiol Community Health.

[11] Deng J, Zhou F, Hou W (2022). Prevalence of mental health symptoms in children and adolescents during the COVID-19 pandemic: a meta-analysis. Ann Ny Acad Sci.

[12] Schnetzer S, Hurrelmann K (2022). Jugend in Deutschland – Trendstudie Winter 2022/23. Die Wohlstandsjahre sind vorbei: Psyche, Finanzen, Verzicht.

[13] Schnetzer S, Hurrlemann K (2022). Trendstudie: Jugend in Deutschland im Dauerkrisenmodus – Klima, Krieg, Corona.

[14] Riley AW (2004). Evidence that school-age children can self-report on their health. Ambul Pediatr.

[15] Choi BCK (2012). The past, present, and future of public health surveillance. Scientifica.

[16] Health ZP (2021) Eine Public-Health-Strategie für Deutschland. In, p 5. https://zukunftsforum-public-health.de/wp-content/uploads/2021/03/ZfPH_PH_Strategie_Policy-Paper.pdf. Zugegriffen: 1. Mai 2023

[17] Thom J, Mauz E, Peitz D (2021). Aufbau einer Mental Health Surveillance in Deutschland: Entwicklung von Rahmenkonzept und Indikatorenset.

[18] Otto C, Reiss F, Voss C (2021). Mental health and well-being from childhood to adulthood: design, methods and results of the 11-year follow-up of the BELLA study. Eur Child Adolesc Psychiatry.

[19] Ravens-Sieberer U, Erhart M, Devine J (2022). Child and adolescent mental health during the COVID-19 pandemic: results of the three-wave longitudinal COPSY study. J Adolesc Health.

[20] Ottova V, Hillebrandt D, Kolip P (2012). Die HBSC-Studie in Deutschland – Studiendesign und Methodik. Gesundheitswesen.

[21] Ravens-Sieberer U, Gosch A, Erhart M (2006). The Kidscreen questionnaires: quality of life questionnaires for children and adolescents: handbook.

[22] Cantril H (1965). The pattern of human concerns.

[23] Goodman R (1997). The strengths and difficulties questionnaire: a research note. J Child Psychol Psychiat.

[24] Woerner W, Becker A, Friedrich C, Rothenberger A, Klasen H, Goodman R (2002). Normierung und Evaluation der deutschen Elternversion des Strengths and Difficulties Questionnaire (SDQ): Ergebnisse einer repräsentativen Felderhebung. Z Kinder Jugendpsychiatr Psychother.

[25] Birmaher B, Khetarpal S, Brent D (1997). The screen for child anxiety related emotional disorders (SCARED): scale construction and psychometric characteristics. J Am Acad Child Adolesc Psychiatry.

[26] Birmaher B, Brent DA, Chiappetta L, Bridge J, Monga S, Baugher M (1999). Psychometric properties of the screen for child anxiety related emotional disorders (SCARED): a replication study. J Am Acad Child Adolesc Psychiatry.

[27] Weissman MM, Orvaschel H, Padian N (1980). Children’s symptom and social functioning self-report scales comparison of mothers’ and children’s reports. J Nerv Ment Dis.

[28] Barkmann C, Erhart M, Schulte-Markwort M, BELLAstudyGroup (2008). The German version of the Centre for Epidemiological Studies Depression Scale for Children: psychometric evaluation in a population-based survey of 7 to 17 years old children and adolescents—results of the BELLA study. Eur Child Adolesc Psychiatry.

[29] Fendrich M, Weissman MM, Warner V (1990). Screening for depressive disorder in children and adolescents: validating the Center for Epidemiologic Studies Depression Scale for Children. Am Epidemiol Rev.

[30] HBSC-Studie Deutschland (2015) Health Behaviour in School-aged Children 2013/14 – Faktenblatt „Methodik der HBSC-Studie“. In, p 6. https://www.gbe-bund.de/pdf/faktenbl_methodik_2013_14.pdf. Zugegriffen: 16. Jan. 2023

[31] HBSC-Studie Deutschland (2020) Health Behaviour in School-aged Children – Faktenblatt „Methodik der HBSC-Studie“ Studie (AutorInnen Moor, Hinrichs, Heilmann, Richter). In, p 6. https://www.gbe-bund.de/pdf/faktenbl_methodik_2017_18.pdf. Zugegriffen: 16. Jan. 2023

[32] Ravens-Sieberer U, Erhart M, Rajmil L (2010). Reliability, construct and criterion validity of the KIDSCREEN-10 score: a short measure for children and adolescents’ well-being and health-related quality of life. Qual Life Res.

[33] Ravens-Sieberer U, Ottova V, Hillebrandt D, Klasen F, HBSC-Team Deutschland (2012). Gesundheitsbezogene Lebensqualität und psychische Gesundheit von Kindern und Jugendlichen in Deutschland: Ergebnisse aus der deutschen HBSC-Studie 2006–2010. Gesundheitswesen.

[34] Wunsch K, Nigg C, Niessner C (2021). The impact of COVID-19 on the interrelation of physical activity, screen time and health-related quality of life in children and adolescents in Germany: results of the Motorik-Modul study. Children.

[35] Vogel M, Meigen C, Sobek C (2021). Well-being and COVID-19-related worries of German children and adolescents: A longitudinal study from pre-COVID to the end of lockdown in Spring 2020. JCPP Adv.

[36] Theuring S, van Loon W, Hommes F (2022). Psychosocial wellbeing of schoolchildren during the COVID-19 pandemic in Berlin, Germany, June 2020 to March 2021. Int J Environ Res Public Health.

[37] Zok K, Roick C (2022) Auswirkungen der Covid-19-Pandemie auf die psychische Gesundheit von Kindern. In: WldO-monitor. p 1–12. https://www.wido.de/fileadmin/Dateien/Dokumente/Publikationen_Produkte/WIdOmonitor/wido_monitor_1_2022_pandemiebelastung_kinder.pdf. Zugegriffen: 16. Jan. 2023

[38] Kaman A, Erhart M, Devine J (2023). Two years of pandemic: the mental health and quality of life of children and adolescents—findings of the COPSY longitudinal study. Dtsch Arztebl Int.

[39] Ravens-Sieberer U, Kaman A, Erhart M (2021). Quality of life and mental health in children and adolescents during the first year of the COVID-19 pandemic: results of a two-wave nationwide population-based study. Eur Child Adolesc Psychiatry.

[40] Meade T, Dowswell E (2015). Health-related quality of life in a sample of Australian adolescents: gender and age comparison. Qual Life Res.

[41] Michel G, Bisegger C, Fuhr DC, Abel T, Kg T (2009). Age and gender differences in health-related quality of life of children and adolescents in Europe: a multilevel analysis. Qual Life Res.

[42] Ravens-Sieberer U, Torsheim T, Hetland J (2009). Subjective health, symptom load and quality of life of children and adolescents in Europe. Int J Public Health.

[43] Moore G, Anthony R, Angel L (2022). Mental health and life satisfaction among 10–11-year-olds in Wales, before and one year after onset of the COVID-19 pandemic. BMC Public Health.

[44] Rajmil L, Hjern A, Boran P, Gunnlaugsson G, Kraus de Camargo O, Raman S (2021). Impact of lockdown and school closure on children’s health and well-being during the first wave of COVID-19: a narrative review. BMJ Paediatr Open.

[45] Blackwell CK, Mansolf M, Sherlock P (2022). Youth well-being during the COVID-19 pandemic. Pediatrics.

[46] Chen X, Cai Z, He J, Fan X (2020). Gender differences in life satisfaction among children and adolescents: a meta-analysis. J Happiness Stud.

[47] Aymerich M, Casas F (2020). A contextualized measure of Overall Life Satisfaction among adolescents: differences by gender. Child Ind Res.

[48] Schlack R, Neuperdt L, Junker S (2022). Veränderungen der psychischen Gesundheit in der Kinder- und Jugendbevölkerung in Deutschland während der COVID-19-Pandemie – Ergebnisse eines Rapid Reviews.

[49] Ma L, Mazidi M, Li K (2021). Prevalence of mental health problems among children and adolescents during the COVID-19 pandemic: a systematic review and meta-analysis. J Affect Disord.

[50] Racine N, McArthur BA, Cooke JE, Eirich R, Zhu J, Madigan S (2021). Global prevalence of depressive and anxiety symptoms in children and adolescents during COVID-19: a meta-analysis. JAMA Pediatr.

[51] Peter F, Dohm L, Krimmer M (2022). Psychische Konsequenzen der Klimakrise: Mehrfachbetroffenheit von Kindern und Jugendlichen angesichts sich verändernder Lebensbedingungen. Monatsschr Kinderheilkd.

[52] Hickman C, Marks E, Pihkala P (2021). Climate anxiety in children and young people and their beliefs about government responses to climate change: a global survey. Lancet Planet Health.

[53] Niessen P, Peter F (2022). Emotionale Unterstützung junger Menschen in der Klimakrise. Klimakrise, sozialökologischer Kollaps und Klimagerechtigkeit. Spannungsfelder für Soziale Arbeit.

[54] Ethikrat D (2022) Pandemie und psychische Gesundheit Aufmerksamkeit, Beistand und Unterstützung für Kinder, Jugendliche und junge Erwachsene in und nach gesellschaftlichen Krisen. Ad-Hoc-Empfehlungen. https://tinyurl.com/3v8zn5ah. Zugegriffen: 16. Jan. 2023

[55] ExpertInnenrat der B (2022) 7. Stellungnahme des ExpertInnenrates der Bundesregierung zu COVID-19 Zur Notwendigkeit einer prioritären Berücksichtigung des Kindeswohls in der Pandemie. https://www.bundesregierung.de/breg-de/bundesregierung/bundeskanzleramt/corona-expertinnenrat-der-bundesregierung. Zugegriffen: 1. Juni 2023

[56] coverCHILD COVID-19 Forschungsplattform für Kinder und Jugendliche. https://coverchild.de/. Zugegriffen: 16. Jan. 2023

