# Supplementary material for: Epidemiology of mental well-being in childhood and adolescence. Results from three epidemiological studies before and during the COVID-19 pandemic
Source: Bundesgesundheitsblatt Gesundheitsforschung Gesundheitsschutz. 2023 May 30;66(7):727–35. [Article in German] doi: 10.1007/s00103-023-03720-5 (PMC10227816; doi:10.1007/s00103-023-03720-5)
Supplement: Supplementary file 1 [file 103_2023_3720_MOESM1_ESM.pdf]

Epidemiologie seelischen Wohlbefindens von Kindern und Jugendlichen in Deutschland. Ergebnisse aus drei Studien vor und während der COVID-19-Pandemie

AutorInnen: Franziska Reiß[1], Anne Kaman[1], Ann-Kathrin Napp[1], Janine Devine[1], Lydia Y. Li[1], Lisa Strelow[1], Michael Erhart[1,2,3], Heike Hölling [4], Robert Schlack [4], Ulrike Ravens-Sieberer[1]

Instituts- und Klinikangaben

[1] *Universitätsklinikum Hamburg-Eppendorf, Zentrum für Psychosoziale Medizin, Klinik für Kinder- und Jugendpsychiatrie, -psychotherapie und -psychosomatik, Hamburg, Deutschland*

[2] *Alice Salomon Hochschule, Berlin, Deutschland*

[3] *Apollon Hochschule der Gesundheitswirtschaft, Bremen, Deutschland*

[4] *Robert Koch-Institut, Berlin, Deutschland*

Korrespondenzadresse

Prof. Dr. Ulrike Ravens-Sieberer

Universitätsklinikum Hamburg-Eppendorf

Zentrum für Psychosoziale Medizin, Klinik für Kinder- und Jugendpsychiatrie,

-psychotherapie und -psychosomatik, Forschungssektion Child Public Health

Martinistraße 52, 20246 Hamburg

E-Mail: ravens-sieberer@uke.de

Telefon: 040 7410-52992

Tabelle Z1. Soziodemographische Daten der BELLA-Studie, COPSY-Studie und HBSC-Studie.

	**BELLA Basis 2003-2006**	**BELLA Welle 3: 2009-2012**	**BELLA IV 2014-2017**	**COPSY I Mai-Jun. 2020**		**COPSY II Dez. 2020-Jan. 2021**		**COPSY III Sep.-Okt. 2021**		**COPSY IV Feb. 2022**		**COPSY V Sep -Okt. 2022**		**HBSC 2002**	**HBSC 2006**	**HBSC 2010**	**HBSC 2014**	**HBSC 2018**
				**EB**	**SB**	**EB**	**SB**	**EB**	**SB**	**EB**	**SB**	**EB**	**SB**					
N	2.863	2.814	1.580	1.586	1.040	1.625	1.073	1.618	1.173	1.668	1.119	1.701	1.085	5.650	7.274	5.005	5.961	4.347
**Geschlecht**																		
Mädchen	48,8%	51,3%	48,4%	50,0%	51,1%	49,7%	51,9%	51,7%	51,0%	51,4%	50,7%	51,2%	50,0%	50,7%	49,6%	51,5%	49,1%	53,0%
Jungen	51,2%	48,7%	51,6%	49,9%	48,8%	50,1%	48,9%	47,8%	48,4%	48,2%	49,1%	48,2%	49,3%	49,3%	50,4%	48,5%	50,9%	47,0%
divers	n.e.	n.e.	n.e.	0,1%	0,1%	0,1%	0,2%	0,5%	0,6%	0,5%	0,7%	0,5%	0,7%	n.e.	n.e.	n.e.	n.e.	n.e.
**Alter** *M* (*SD*)	*12.29 (3.19)*	*11.35 (4.62)*	12.73 (3.21)	12.25 (3.30)	14.33 (1.86)	12.59 (3.21)	14.35 (2.17)	13.27 (3.24)	14.74 (2.41)	13.07 (3.77)	15.08 (2.60)	13.09 (3.80)	15.12 (2.62)	13.49 (1.71)	13.44 (1.67)	13.35 (1.69)	13.45 (1.65)	13.41 (1.68)
**Altersgruppen[1]**																		
7–10 Jahre	33,2%	28,1%	34,5%	35,8%	n.e.	31,5%	n.e.	24,8%	n.e.	30,3%	n.e.	30,3%	n.e.	n.e.	n.e.	n.e.	n.e.	n.e.
11–13 Jahre	26,1%	12,0%	25,9%	26,9%	41,9%	20,4%	29,5%	27,7%	35,7%	22,9%	32,6%	22,9%	32,2%	69,1%	64,7%	66,9%	64,4%	64,8%
14–17 Jahre	40,8%	31,5%	39,6%	37,3%	59,1%	48,1%	70,5%	47,4%	64,3%	46,8%	67,4%	46,8%	67,8%	30,9%	35,3%	33,1%	35,1%	35,2%
**Bildung Eltern**																		
hoch	27,3%	39,1%	26,0%	24,1%	26,6%	24,9%	27,0%	27,2%	28,5%	30,1%	27,1%	31,8%	27,4%	n.e.	n.e.	n.e.	n.e.	n.e.
mittel	53,5%	54,0%	50,0%	55,7%	52,7	56,1%	54,2%	57,1%	56,5%	55,7%	56,6%	53,5%	54,9%	n.e.	n.e.	n.e.	n.e.	n.e.
niedrig	18,8%	6,9%	22,1%	18,2%	18,5%	17,8%	17,6%	15,8%	15,0%	14,1%	16,3%	14,7%	17,8%	n.e.	n.e.	n.e.	n.e.	n.e.
**Migration**																		
ja	12,1%	5,9%	13,3%	16,0%	15,5%	16,4%	15,8%	18,2%	17,3%	17,5%	16,5%	17,3%	17,3%	n.e.	n.e.	n.e.	27,3%	35,3%
nein	87,9%	87,9%	86,1%	84,0%	84,5%	83,6%	84,2%	81,8%	82,7%	82,5%	83,5%	82,7%	82,7%	n.e.	n.e.	n.e.	72,7%	64,7%

Anmerkungen. [1]Anteil bezogen auf die Gesamtstichprobe, Bildung der Eltern nach CASMIN-Klassifikation; Migration = Migrationshintergrund; EB = Elternbericht; SB = Selbstbericht; n.e. = nicht erhoben.
